# Effectiveness of an educational program on knowledge of selected common pregnancy discomforts among pregnant mothers in Al-Ahsa, Saudi Arabia

**DOI:** 10.18332/ejm/224230

**Published:** 2026-07-03

**Authors:** Noura Mohammed Alsubaie, Ibtesam Omar Jahlan

**Affiliations:** 1 MSN, Training Department, AlAhsa Health Cluster, Al-Ahsa, KSA; 2 Maternal and Child Health Nursing Department, College of Nursing, King Saud University

**Keywords:** pregnancy, pregnant women, discomforts, knowledge, self-management

## Abstract

**INTRODUCTION:**

Insufficient understanding of pregnancy's common discomforts and their management has been seen among pregnant women. Thus, antenatal care should include comprehensive education on common pregnancy discomforts, delivered by maternity nurses, who play a crucial role in educating pregnant mothers. The aim of the study is to evaluate the effectiveness of an educational program in improving pregnant mothers' knowledge and management options regarding common pregnancy discomforts in Al-Ahsa, Saudi Arabia.

**METHODS:**

A quasi-experimental, one-group pretest and posttest design was conducted in Al-Ahsa province, Saudi Arabia, among 131 pregnant mothers from the Maternity and Children's Hospital and primary health centers. Data were collected via a self-completed questionnaire in February 2024. Educational sessions were conducted as the study intervention. A paired t-test was used with p<0.001. Primary outcomes revealed increased knowledge in post-testing, supporting the effectiveness of the educational program.

**RESULTS:**

Using a pre-test as baseline knowledge, the study showed that 46.6% of participants possessed a moderate knowledge level regarding common pregnancy discomforts; meanwhile, 66.4% reported a good knowledge level regarding self-management practice. Following the educational intervention, the mean knowledge score increased significantly from 37.0 to 48.3 (mean difference=11.2, p<0.001). Similarly, the mean practice score increased significantly from 36.8 ± 7.0 to 42.6 ± 5.3 (mean difference=5.8, p<0.001).

**CONCLUSIONS:**

The study found that education significantly enhanced mothers’ knowledge and self-management of pregnancy discomforts, providing evidence supporting the implementation of education as an effective approach in prenatal care.

## INTRODUCTION

Pregnancy represents a unique experience for each mother, involving emotions of pleasure and excitement. However, it also involves physiological and psychological changes due to hormonal changes to accommodate uterine and fetal development, causing common pregnancy discomforts that could affect the mother’s emotions, wellbeing, and daily activities^1^. High prevalence of common pregnancy discomforts has a significant impact on pregnant mothers and their families^2^. Additionally, it is possibly leading to increased healthcare visits and, in serious situations, hospitalization^2,3^.

Common pregnancy discomforts include anatomical, physiological, and biochemical changes. They vary from mother to mother and also by trimester. For example, in early pregnancy, the most common pregnancy discomfort may start with fatigue, nausea, vomiting, frequency of micturition, constipation, or vaginal discharge. Thereafter, toward the end of the pregnancy, the mother may experience backache, leg cramps, edema, frequency of micturition, fainting, insomnia, or varicosities^4^.

Initially, maternal health issues predominantly centered on obstetric complications and psychiatric disorders. However, a comprehensive approach now considers discomforts that may affect pregnancy experiences^5^. It is equally necessary to provide the mother with education regarding the physiological causes of these discomforts and self-care methods that can effectively alleviate them and empower women to effectively manage their pregnancy journey and ensure the wellbeing and comfort of both them and the fetus^4,6^. In addition to common pregnancy discomforts, pregnant mothers also need to be aware of warning signs and symptoms that may indicate serious pregnancy complications such as severe vomiting (hyperemesis gravidarum), epigastric pain, excessive vomiting, difficulty breathing, and edema related to pre-eclampsia^7^. Therefore, maternity nursing has a crucial role in focusing on educating them about common pregnancy discomforts through various methods^5^.

The aim of this study was to evaluate the effectiveness of an educational program in improving pregnant mothers’ knowledge in Al-Ahssa, Saudi Arabia, about common pregnancy discomforts and appropriate options to alleviate them. Our aim was to evaluate the knowledge and self-management practice scores of pregnant women before and after the educational intervention.

## METHODS

### Research design

This study utilizes a quasi-experimental, one-group pretest–posttest design to assess the effectiveness of an educational program in improving the mothers’ knowledge level. The schematic diagram that applies to this study is as follows: Observation or measurement (pretest measurement); Intervention (educational session); and Observation or measurement (posttest measurement).

### Study setting

The study was conducted at the Maternity and Children Hospital (MCH) in Al-Ahsa, managed by the Al-Ahsa Health Cluster (AHC) under the Ministry of Health (MOH). It was decided to select MCH because it is recognized as a prominent public hospital in the region. In addition to the three Primary Health Centers (PHCs) that are affiliated with the MCH.

### Sampling, study population, and sample size

A non-probability convenience sampling technique was adopted to select the sample for this study. The target population for this study was pregnant mothers at the selected sites. The population readily available for study was pregnant mothers presenting in OPDs and antenatal clinics in the selected PHCs at the time of data collection. The study population was calculated by counting the monthly visits of mothers in the chosen setting, specifically, for the two-month period that was planned for data collection as provided by MCH; therefore, the population size for this study was determined, and the sample size was determined to be 131 mothers. The sample size was calculated using the Raosoft website (Sample Size Calculator; Raosoft Inc.). A number of inclusion criteria were considered when selecting the sample: 1) aged 18–35 years; 2) mothers with low-risk pregnancies; 3) Arabic-speaking mothers; and 4) being able to use an electronic device with an internet connection. Also, a number of exclusion criteria were taken into account: 1) mothers with a high-risk pregnancy representing a medical, surgical, or obstetrical condition such as antepartum hemorrhage, gestational diabetes mellitus (GDM), or pregnancy-induced hypertension; and 2) mothers aged <18 years or >35 years.

### Data collection methods

Data were collected using a structured self-administered questionnaire, with permission from its original author, that employed as pretest and posttest which included items related to common pregnancy discomforts according to the most often reported by World Health Organization (WHO), National Institute for Health and Care Excellence (NICE), and Saudi Ministry of Health (MOH)^8-10^, such as nausea, back pain, heartburn, and leg cramps and self-management options to alleviate them. The tool was validated and reliable, with Cronbach’s alpha scores of 0.95 and 0.95 to ensure reliability and validity, and to identify potential issues^11^.

### Study tools

*Section I: Demographic data*

Description of demographic data of the mother, such as age, education level, occupation, and family income.

*Section II: Obstetric history*

This comprises the number of previous pregnancies; gestational age for the current pregnancy; a description of the current pregnancy discomforts, specifically the starting time of the pregnancy discomforts; and the self-reported intensity of the pregnancy discomforts.

*Section III: Knowledge assessment regarding pregnancy discomforts*

This section aims to evaluate mothers’ knowledge regarding pregnancy discomforts, their causes, and the warning signs associated with them. It contains 22 multiple-choice grid questions presented in tables, which enable the mothers to select one answer in each row. In data analysis, these questions are displayed in an Excel sheet as multiple-choice questions (MCQs), with a total of 63 MCQs.

Section IV: Knowledge assessment regarding the correct management choices of pregnancy discomforts

This section is intended to assess the mother’s knowledge about appropriate methods of relieving pregnancy discomforts. It contains nine multiple-choice grid questions presented in tables, which enable the mothers to select one answer in each row. In data analysis, these questions are displayed in an Excel sheet as MCQs, with a total of 47 MCQs.

### The scoring system

The test consists of 110 items in a grid format: 63 questions in section one focuses on knowledge about pregnancy discomforts, their causes, and warning signs, and 47 questions in section two focuses on practical knowledge in managing pregnancy discomforts. The full mark is 94 points, with 47 points allocated to the knowledge part and 47 points to the practical knowledge section.

Correct answers were granted a score of 1, while incorrect answers or responses of ‘I don’t know’ received a score of 0. The scores were converted to percentages and categorized into three groups: scores <50% indicate a low knowledge level, 50–75% indicate a moderate knowledge level, and scores >75% indicate a good knowledge level.

### Study intervention

The study used a structured educational program to improve the pregnant mother’s knowledge regarding common pregnancy discomforts, their causes, warning signs, and self-management options. The program’s objectives were to provide participants with knowledge of the definitions of common pregnancy discomforts typically experienced during the first, second, and third trimesters; their underlying causes; associated warning signs that require medical attention; and evidence-based self-management strategies.

Educational content developed by the researcher based on relevant maternal health references was delivered through an online session via the Zoom platform. The teaching materials consisted of PowerPoint presentations, supplemented by educational videos and illustrative images to enhance participants’ understanding. A maternal health expert reviewed the content and educational materials prior to implementation.

Each education session lasted 40–45 minutes and was held several times during the data collection period to accommodate participants’ availability. Sessions were conducted in small groups of approximately 6–10 mothers. Participants who were unable to attend the live sessions could also access the recorded sessions.

A pre-test was administered to the participants before the educational session to determine the baseline knowledge and self-management practices. Then, participants were assessed with the same questionnaire as a posttest immediately after the educational session.

### Data collection procedures

The data collection process started in February 2024; it involved three phases, after obtaining verbal permission from participants: a pre-test to evaluate mothers’ current knowledge of common pregnancy discomforts and self-management options, then an online educational session (study intervention), and lastly an immediate posttest after the program to evaluate the improvements in mothers’ knowledge scores.

### Data analysis

For data analysis, SPSS software was used to analyze the data, including descriptive statistics, inferential statistics, the Pearson correlation coefficient (ANOVA), and t-tests to examine the intended aim of this study.

## RESULTS

### Demographic characteristics

The study showed that the majority of mothers (51.9%) were aged 21–25 years, followed by 25.9% in the age group of 26–30 years. Also, regarding education level, the vast majority of participants (80.9%) reported holding a university degree. Regarding occupation, a considerable proportion of participants (74.8%) identified as housewives, while 25.2% reported being employed as workers. In relation to income, the largest group of participants (43.5%) reported a monthly family income of <5000 SAR. Further details are shown in Table 1.

**Table 1:** Sociodemographic characteristics of pregnant mothers participating in a quasi-experimental study evaluating the effectiveness of an educational program on knowledge of common pregnancy discomforts in Al-Ahssa, Saudi Arabia (N=131)

*Characteristics*	*n*	*%*
**Age** (years)		
18–20	4	3.0
21–25	68	51.9
26–30	34	25.9
31–35	25	19.1
**Education level**		
Primary school	1	0.8
Middle school	1	0.8
High school	23	17.6
University	106	80.9
**Occupation**		
Worker	33	25.2
Housewife	98	74.8
**Monthly income** (SAR)		
<5000	57	43.5
5000–10000	49	37.4
10001–15000	16	12.2
>15000	9	6.9

SAR: 1000 Saudi Arabian Riyals about US$270.

### Obstetrical characteristics

The study found that 74.1% of participants experienced moderate discomfort during pregnancy, with 19.1% experiencing severe discomfort. The mean number of previous pregnancies was 2.0, and the mean gestational age for the current pregnancy was 24.9 weeks. Discomforts typically began around the ninth week of gestation. These findings can help healthcare providers provide appropriate support and care during pregnancy, as they can understand the participants’ experiences with common pregnancy discomforts and their timing. Further details are shown in Table 2.

**Table 2:** Obstetrical characteristics of pregnant mothers participating in a quasi-experimental study evaluating the effectiveness of an educational program on knowledge of common pregnancy discomforts in Al-Ahssa, Saudi Arabia (N=131)

*Variable*	*n*	*%*
**Pregnancy discomfort severity**		
Mild	9	6.9
Moderate	97	74.0
Severe	25	19.0
**Number of previous pregnancies,** mean (SD) range	2.0 (1.2) 1–5	
**Gestational age for the current pregnancy** (weeks), mean (SD) range	24.9 (10.7) 4–41	
**The time when discomforts begin to occur is in which week,** mean (SD) range	9.2 (6.3) 1–36	

Pregnancy discomfort severity (mild, moderate, severe) was self-reported by the participants based on their perceived level of symptom severity.

Mothers’ knowledge and practical knowledge levels regarding selected common pregnancy discomforts.

Regarding knowledge, the study revealed that nearly half of the mothers (46.6%) demonstrated an average level of understanding regarding the causes and warning signs of common pregnancy discomforts, while two-thirds (66.4%) reported good practices in managing these discomforts, as shown in Table 3.

**Table 3:** Levels of knowledge and self-management practices regarding common pregnancy discomforts of pregnant mothers participating in a quasiexperimental study evaluating the effectiveness of an educational program on knowledge of common pregnancy discomforts in Al-Ahssa, Saudi Arabia (N=131)

*Variables*	*n*	*%*
**Level of mothers’ knowledge on common pregnancy discomforts** (%)		
Low (<50)	38	29.0
Average (50–75)	61	46.6
Good (>75)	32	24.4
**Levels of practice in managing common pregnancy discomforts** (%)		
Low (<50)	4	3.0
Average (50–75)	40	30.5
Good (>75)	87	66.4

### Effectiveness of an educational intervention on mothers’ knowledge and practical knowledge of common pregnancy discomforts

The study found a significant difference in knowledge about common pregnancy discomforts between the pretest and posttest. Participants had a statistically significant improvement in knowledge ratings post-educational session. The mean score of knowledge was 37.0 (SD=11.5) before the intervention and 48.3 (SD=8.6) after the intervention. A paired t-test showed a significant difference between pre- and post-intervention scores [t(130)= -9.1, p<0.001]. Knowledge scores improved by 11.3 points. Specifically, post-intervention (educational session) knowledge scores were significantly higher than pre-intervention (educational session) scores by an average of 11.2 points.

Furthermore, there was a significant increase in the scores of the practical knowledge after the intervention. Mean practice score increased from 36.8 (SD=7.0) before the intervention to 42.6 (SD=5.3) after the intervention. A paired t-test showed a statistically significant difference between the pre- and post-intervention practice scores [t(130)= -7.7, p<0.001], which is an average gain of 5.8 points.

In summary, the findings indicated that the educational program administered between pre- and post-testing was effective in significantly enhancing mothers’ comprehension of common pregnancy discomforts.

## DISCUSSION

The main finding of this study was that the educational program successfully improved mothers’ knowledge of selected common pregnancy discomforts, as evidenced by higher posttest scores. A number of previous studies align with the current results, providing solid evidence that the educational session and other teaching methods are effective ways to enhance mothers’ comprehension and address pregnancy-related issues.

This study aligns with Gururani et al.^12^, who reported that a planned teaching program significantly improved pregnant women’s understanding of common pregnancy discomforts and their home management. Similarly, Latha and Indira^13^ demonstrated the effectiveness of educational programs, such as the Information, Education and Communication (IEC) package, as reflected in a higher posttest mean score (23.9) compared to the pre-test mean (14.7). Supporting evidence is also provided by Dhanawade^14^, who found an increase in average posttest scores following a structured teaching program, further indicating the success of planned educational interventions. In addition, Hashem et al.^15^ and El-Sarkawy et al.^16^ confirmed that both face-to-face sessions and combined approaches such as educational booklets and classroom lectures were effective in enhancing mothers’ knowledge, as shown by the significant differences between pretest and posttest scores. Additionally, the study of Kumar^17^ revealed that the utilization of the Self-Instructional Module (SIM) successfully improved mothers’ understanding of common pregnancy discomforts and of the several strategies available for addressing them. Collectively, these studies substantiate the effectiveness of diverse educational strategies in improving women’s awareness of pregnancy-related discomforts, a finding consistent with the present study.

Maternity nursing should emphasize promoting the health of pregnant women, so the implications for this study include teaching mothers about common pregnancy discomforts and appropriate ways to alleviate them, using different educational methods such as face-to-face instruction in the antenatal clinic, educational booklets, leaflets, virtual teaching sessions, or group learning. Additionally, an in-service education program should be conducted for nursing personnel to enhance their educational skills so they can educate mothers and correct misinformation about the appropriate care of pregnancy discomforts. Notably, the expansion of maternity and midwifery programs in Saudi Arabia, which includes both undergraduate^18,19^ and postgraduate^20,21^ education as well as Saudi Commission for Health Specialties postgraduate training programs in maternity and midwifery nursing^22^, reflects the growing national focus on this field and will certainly contribute to improving maternity care by enhancing antenatal education for pregnant women and the maternity services provided by qualified and specialized nurses and midwives.

### Limitations

The study has several limitations, including its focus on one secondary hospital and three PHCs in a specific region. The data collection procedure also had limitations, with content review limited to one expert and multiple implementations via Zoom requiring greater attention to contextual content accuracy and session validity. The posttest immediately after the educational session introduced the potential for a memory effect. These limitations will help future researchers improve the quality of findings on pregnant mothers’ education.

## CONCLUSIONS

It was shown that after performing the educational program on common pregnancy discomforts, mothers’ knowledge regarding pregnancy discomforts and their appropriate options to alleviate them will be significantly higher than the mean of the pre-test score. Results showed that mothers exhibited statistically significant improvements in knowledge following participation in the program compared to before. In summary, a fundamental nursing responsibility during the prenatal period is to educate mothers about common pregnancy discomforts and self-management options to enhance their comfort during pregnancy.

## Data Availability

Data sharing is not applicable to this article as no new data were created.
